# Draft Genome Sequence of *Pannonibacter indicus* Strain HT23^T^ (DSM 23407^T^), a Highly Arsenate-Tolerant Bacterium Isolated from a Hot Spring in India

**DOI:** 10.1128/genomeA.00283-17

**Published:** 2017-05-04

**Authors:** Saumya Bandyopadhyay, William B. Whitman, Subrata K. Das

**Affiliations:** a Department of Biotechnology, Institute of Life Sciences, Bhubaneswar, India; b Department of Microbiology, University of Georgia, Athens, Georgia, USA

## Abstract

*Pannonibacter indicus* strain HT23^T^, a highly arsenate-tolerant bacterium, was isolated from a tropical hot spring. The estimated genome is 4.2 Mb with 3,818 protein-coding sequences containing putative genes, some of which are involved in arsenate resistance.

## GENOME ANNOUNCEMENT

*Pannonibacter indicus* strain HT23^T^ is a motile, aerobic, Gram-negative, and rod-shaped bacterium belonging to the class *Alphaproteobacteria*. This bacterium was isolated from a tropical hot spring at Athamallik, Orissa, India (1, 2). Strain HT23^T^ is able to grow in the presence of 500 mM of sodium arsenate in low-phosphate medium. The genome sequence will help to understand the mechanism of arsenic tolerance in this bacterium.

The draft genome of strain HT23^T^ was generated at the DOE Joint Genome Institute (JGI). An Illumina standard shotgun library was constructed and sequenced using the Illumina HiSeq 2000 platform (3), which generated 10,175,130 paired-end reads totaling 1,536.4 Mb. Filtered Illumina reads were assembled using Velvet (4), wgsim, and Allpaths-LG (5). The final draft assembly contained 38 contigs in 36 scaffolds, totaling 4.2 Mb, with an input read coverage of 261.8-fold. The largest and *N*_50_ contigs were 646.4 kb and 216.1 kb, respectively, with a G+C content of 63.5%.

The genome was annotated using the JGI Microbial Annotation Pipeline (6). Genes were identified using Prodigal (7), followed by manual curation using GenePRIMP (8). The tRNAs, rRNAs, and other noncoding RNA genes were identified by searching the genome using the tRNAScan-SE tool (9), rRNA gene models built from SILVA (10), and Infernal (https://www.janelia.org/publication/infernal-10-inference-rna-alignments), respectively. The draft genome sequence has 3,818 candidate protein-coding sequences (CDSs), 50 tRNAs, 7 rRNAs, 13 other RNAs, and 3 clustered regularly interspaced short palindromic repeats (CRISPRs).

### Accession number(s).

This whole-genome shotgun project has been deposited at DDBJ/EMBL/GenBank under the accession number LIPT00000000. The version described in this paper is the first version, LIPT01000000.
